# Characterization of a simple potentiometric graphite based sensor and its effective applicability in sensitive and selective determination of Cu(II) ion in vegetable foliar, real water and pharmaceutical samples

**DOI:** 10.1038/s41598-025-25089-y

**Published:** 2025-11-18

**Authors:** Menna A. Gaber, Gehad G. Mohamed, M. M. Omar, Aya E. Ali

**Affiliations:** 1 Chemistry Department, Faculty of Science, Cairo University, Giza, 12613 Egypt; 2 Nanoscience Department, Basic and Applied Sciences Institute, Egypt-Japan University of Science and Technology, New Borg El Arab, Alexandria 21934 Egypt

**Keywords:** Graphite sensor, Schiff base, Cu(II) ion, Standard addition method, Scanning electron microscope (SEM), Energy dispersive x-ray (EDX), Chemistry, Environmental sciences, Materials science

## Abstract

The importance of Cu(II) in environmental, biological, and industrial systems makes it crucial to develop a highly selective and sensitive analytical methodology for its determination. Herein, a graphitic based sensor modified with a Schiff base, namely 2-(((3-aminophenyl) imino) methyl) phenol was developed and applied. A broad concentration range (1 × 10^−7^- 1 × 10^−1^) mol L^−1^ was covered by the electrode’s linear response to Cu(II), which had a 29.571 ± 0.8 mV decade^−1^ Nernstian slope. It was highly reproducible (inter and intraday RSDs%=0.94–2.12), with a response time of approximately 15 s and pH working rang was from 3.5 to 6.5. and had a two-month lifespan. With this modified sensor, 5.0 × 10^−8^ mol L^−1^ was the detection limit and1.65 × 10^−7^ mol L^−1^ was the limit of quantification. Over a wide range of metal ions, the suggested electrode exhibited very good selectivity for Cu(II) applying separate solution method(SSM), fixed interference method (FIM) and matched potential method (MPM). Cu(II) levels in Several samples like spiked water samples, Hairvogine multivitamin and Nutrifol vegetable foliar were accurately determined potentiometrically by either direct, or standard addition methods using this chemically altered carbon paste electrode giving a comparable results with atomic absorption spectroscopy (AAS) technique. Scanning electron microscope (SEM) combined with energy dispersive X-ray (EDX) were utilized for morphological analysis of sensors’ reactive surface combined with elemental analysis as well.

## Introduction

Since its superior electrical and thermal conductivities, and flexibility, copper is a significant industrial metal that is employed in many different sectors^1,2^. Copper slag was once frequently considered industrial waste and was either used directly as building material or tossed of in landfills, resulting in a large waste of resources. The recovery of copper from secondary resources has received a lot of interest lately due to the advantages of the hydrometallurgical extraction method, which includes affordable prices as well as reduced energy consumption^3^. Therefore, investigating copper’s dissolution is crucial. Although copper (Cu) is found in all bodily tissues, it primarily accumulates in the liver and is also found in lesser quantities in the brain, heart, kidney, and muscles^4^. Copper is known for its antioxidant and pro-oxidant capabilities. As it is needed for the correct operation of numerous basic and fundamental enzymes and also for the synthesis of haemoglobin, myelin, and melanin, copper is a key component of human metabolism. Not just how important it functions as substrates for biomolecules’ structural and metabolic processes^5^. The impact of heavy metal pollution on ecosystems and people has received a lot of attention during the past forty years. Both natural and man-made sources, such as a range of industrial resources like mining sites, foundries and smelters, combustion byproducts, and traffic, can release heavy metals into the atmosphere^6–8^. Specifically, heavy metal ions have the ability to interact with proteins in the body to form robust and stable chemical bonds, which can disrupt a person’s regular metabolic functions. Cu(II) can be toxic at higher concentration level as it causes gastrointestinal illness, Wilson disease, hypoglycemia and dyslexia.

One of the most urgent needs of the day is the implementation of targeted investigations on monitoring the concentrations of heavy metals in environmental solutions in order to assure human safety. Several analytical tools have been researched and developed in recent years to determine the heavy-metal ions (such as Cu(II), Fe(III), Cd(II), Pb(II), and Ag(I)) in solutions^9,10^. Low cost, good reproducibility, and low detection limits make ion-selective electrodes (ISEs) one of the most successful devices. Electrochemical sensors and biosensors have a wide range of applications in determination of many chemical species^11–17^.

Potentiometric detection utilizing ISEs has the advantages of being simple, rapid, relatively selective, non-destructive, and environmentally benign. ISEs had a short duration and unreliable responses when they started out as liquid contact electrodes. Solid contact electrodes were subsequently created as a result. These glassy carbon and coated wire solid phase electrodes, among others, had a number of disadvantages, like the formation of interior water films. As a result, the electrode’s surface changes and the rate at which electrons transfer over it slows^18^. In order to improve response behaviours, membrane electrodes have undergone substantial improvement and modification in the past few years^19–21^. In order to use carbon paste electrodes (CPEs) as functional electrodes in electrochemical sensing applications, researchers are focusing more on CPEs lately. Surface reproducibility, stability, and renewability are just a few of the desirable qualities that make CPEs attractive material for working electrodes^22–24^. In fields like pharmacological, biological, and environmental studies, CPEs are being utilized increasingly frequently because of their low cost in comparison to other materials. Improvements of CPE’s physical or chemical nature can enhance its adsorption capacity, sensitivity, and selectivity^25,26^. To generate an effective electrode with relevant sensing capabilities for users is the aim of changing a CPE matrix. Electrocatalytic activities at the CPE interface will generally increase following alterations. This may be because of the increased surface area, which provides improved current sensitivity for electron transfer reactions. Conductive substrates changed with electroactive thin films, monolayers, or thick coatings can be used to chemically modify CPE.

Several methods have been reported for Cu(II) ion determination besides the potentiometric technique as atomic absorption spectrometry (AAS), inductively coupled plasma (ICP), anodic stripping voltammetry (ASV), amperometric and flame photometry^27–31^. However, the challenge of this work was introducing a novel CPE potentiometric method that have superior criteria over the previously reported methods for Cu(II) ion determination regarding simplicity, renewability, low cost, sensitivity, selectivity, fast response, no need for sample pretreatment and wide working pH range.

Various chemical and inorganic substances have been employed as ionophores for the selective detection of Cu(II) ions in the literature throughout the past few decades^9,12,32–37^. However, many of these reported electrodes have some disadvantages like short shelf time, low sensitivity, serious interference from other metal ions, long time of response, and a limited working range of pH. Therefore, development of more effective sensors with enhanced features is a challenge and in turn it will improve the application of ISEs in routine analysis.

**Fig. 1:**
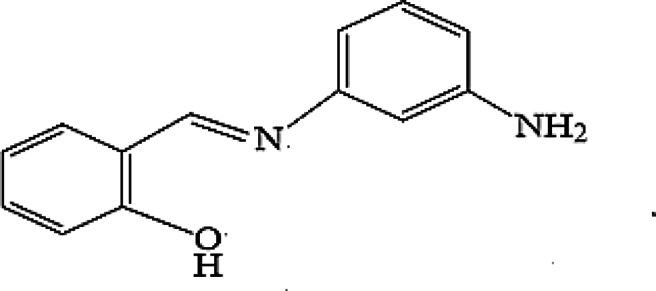
Structure of 2-(((3-aminophenyl) imino) methyl) phenol Schiff base ligand.

2-(((3-aminophenyl) imino) methyl) phenol Schiff base ligand (Fig. 1) was reported to form stable complexes with transition metal ions^38^. This Schiff base is a tridentate ligand with phenolic, amino and azomethine bonding groups that can form stable 1:1 chelates with transition metal ions. In this work we have studied the electrochemical application of this modifier in a CPE matrix to selectively detect Cu(II) ion and the results showed satisfactory selectivity coefficient values, wide pH working range, thermal stability and fast response time. Low standard and relative standard deviations, high % recovery rates, and acceptable low limits of detection and quantification were reported. The potentiometric method showed no significant difference with the official method according to the calculated F- and t-test values. The results obtained encourage to apply this potentiometric method in routine analysis measurement of Cu(II) ions in different samples without the needs for pretreatment or sophisticated extraction techniques.

## Experimental

### Materials

In this investigation, analytical grade chemicals were implemented. All of the tests used distilled water. Merck provided the copper sulphate [CuSO_4_.5H_2_O] that was used. *o*-Nitrophenyl octyl ether (*o*-NPOE) was purchased from Fluka while dioctyl phthalate (DOP), dibutyl phthalate (DBP) were purchased from BDH. Tricresyl phosphate (TCP), diheptyl phthalate (DHP) and graphite powder (synthetic 1–2 μm) were purchased from Sigma Aldrich. Chloride salts of manganese, cadmium, zinc, nickel, calcium, magnesium, lead, barium, cobalt, chromium and aluminium used as interfering materials were purchased from El Nasr Company.

### Apparatus

The generated graphite sensors were employed to monitor potential using a double-junction reference electrode composed of silver-silver chloride (HANNA, HI 5311). To monitor pH, a Jenway 3505 pH meter was utilized. The energy dispersive X-ray analyzer (EDX) and the National Research Centre Quanta FEG250 scanning electron microscope, both made in Egypt, were used for surface examination. A Perkin-Elmer 1650 spectrometer (4000–400 cm^−1^) was used to study the FT-IR spectra of potassium bromide pellets at Cairo University in Egypt’s Microanalytical Centre.

### Procedures

#### Synthesis of the schiff base ligand (HL)

**Scheme 1:**
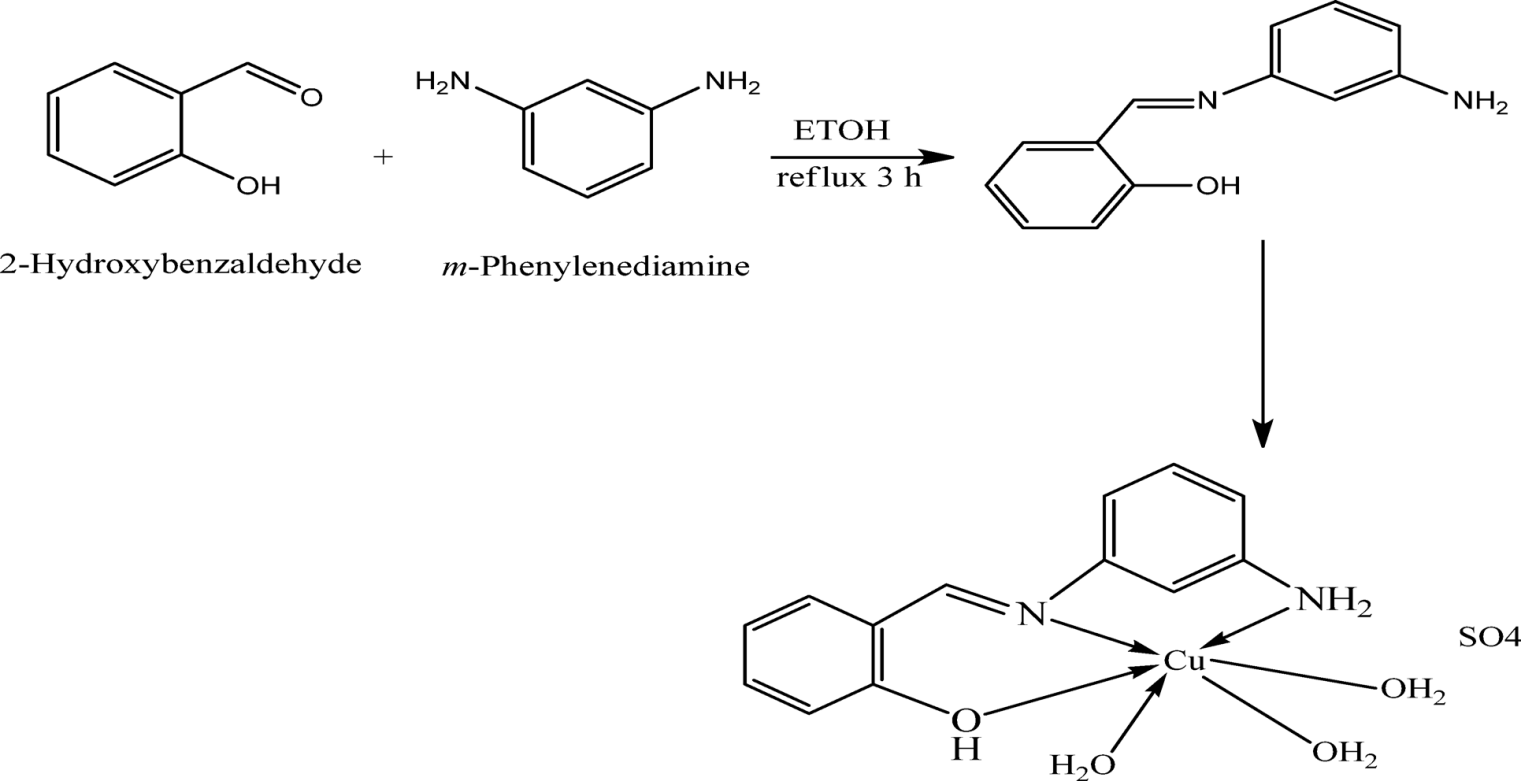
Preparation of ionophore and its suggested Cu(II) complex^38^.

Using ethanol as a solvent, m-phenylenediamine (129.4 mmol, 14 g) and 2 hydroxybenzaldehyde (129.4 mmol, 15.8 g, 13.5 ml) underwent a condensation reaction to create Schiff base ligand (HL), as reported^38^. A yellowish green solid substance was isolated from the resultant mixture after it was swirled under reflux for almost three hours. After filtering, recrystallising, and washing with diethyl ether, it was vacuum-dried^38^. Yield 50.70%; solid yellowish green; m.p. 130 °C. FT-IR (cm^−1^): phenolic v(OH) 3431, azomethine ν(C = N) 1613, ν(C‐N) 1396, ν(C‐O) phenolic 1274. ^1^H-NMR (300 MHz, DMSO‐d6, δ, ppm): 9.10 (s, H, OH phenolic), 8.86 (s, H, CH = N), 6.54–7.69 (m, 8 H, Ar H), 4.23 (s, 2 H, NH_2_). λ_max_ (cm^−1^): 30,030 n–π*, 46,082 and 40,650 π–π* (44) (Scheme 1).

#### Preparation and characterization of MCPE

In a mortar, 250 mg of pure graphite powder, 5–20 mg of the produced ionophore, and 0.1 mL of plasticizer (*o*-NPOE, DOP, TCP, DBP, or DHP) were thoroughly mixed. Before being used, the modified paste was stored in distilled water for twenty-four hours. It was then filled into a Teflon holder that served as the electrode body. A stainless-steel rod was pushed into the holder’s centre to allow for electrical contact. The shiny new surface was achieved by polishing a fresh carbon paste surface on filter paper after pushing the stainless-steel screw forward^16,17^.

For the proposed CPE surface’s characterization, the energy dispersive X ray analyzer (EDX) and National Research Centre Quanta FEG250 scanning electron microscope, both made in Egypt, were used for surface examination before and after interaction with Cu(II) ions. A Perkin-Elmer 1650 spectrometer (4000–400 cm^−1^) was used to study the FT-IR spectra of potassium bromide pellets at Cairo University in Egypt’s Microanalytical Centre.

#### Electrochemical/potentiometric measurements and calibration

The calibration of the prepared sensor under investigation (250 mg graphite, 10 mg of prepared Schiff base ligand and 100 mg TCP) was established by immersion of carbon paste electrode in conjugation with a double-junction reference electrode composed of silver-silver chloride (HANNA, HI 5311) in a 25 mL beaker containing 5.0 mL aliquots of 1.0 × 10^−7^ – 1.0 × 10– ^1^ mol L^−1^ of Cu(II) solution. The response of the sensors for copper ion was examined by measuring electromotive force (emf) of the following electrochemical cell: Ag | AgCl |satd. KCl || sample solution | MCPE. The obtained response obeys Nernstian equation: E = E^0^ +(2.030 RT/nF) log [Cu (II)] where, E is potential, E^0^ is a constant characteristic of a particular ISE, R is the gas constant (8.314 J/K.mol), T is the temperature (in K), n is the charge of the ion and F is Faraday constant (96,500 C/mol). A 25 mL beaker containing a 10 mL aliquot of buffered Cu(II) solution, buffered at pH = 4.5 using acetate buffer, with concentrations ranging from 1 × 10^−7^ to 1 × 10^−1^ mol L^−1^ was used to calibrate the Cu(II) graphite selective electrode while swirling steadily. The obtained stabilised potentials at 25 ^◦^C ± 1 were plotted against log [Cu (II)] in order to investigate the unknown Cu(II) ion concentration.

#### Selectivity analysis

As stated before^16,17,39^, the separate solution method (SSM), which linked the potentials of the two solutions using the Debye-Huckel equation, was used to do selectivity analysis in separate solutions. The fixed interference approach (FIM) was used to analyse the study in a mixed solution setting; the concentration of the Cu(II) ion was varied from 1 × 10^−7^ to 1 × 10^−1^ mol L^−1^, and the modified Cu(II) sensor and reference electrode were placed in constant 1 × 10^−3^ mol L^−1^ concentration of interfering ions. The acquired calibration plots’ detection limits were used to evaluate the coefficients of selectivity. Matched Potential Method (MPM) was suggested for figuring out an ion-selective electrode’s selectivity coefficient. After introducing either the primary ion or the secondary ion, potential changes are monitored using a reference target ion solution. To achieve the same potential shift as obtained for a fixed additional concentration of the primary ion, increasing concentrations of the secondary ion are added. The selectivity coefficient for this potential change is represented by the ratio of primary to secondary ion concentrations^40^. The fundamental benefit of MPM is its independence from the Nicolsky-Eisenmen equation, which has drawbacks such as non-Nernstian interfering ion behaviour and unequal primary and interfering ion charges, as in SSM and FIM.

####  Samples analysis

Each sample was spiked to produce three concentrations of Cu(II) (0.635 × 10^−3^, 6.350 × 10^−3^ and 63.50 × 10^−3^ mg mL^−1^) after the water samples’ pH was brought down to the desired level of 4 using NaOH or HNO_3_, making up El-Giza tap water (sample 1) and underground water (sample 2). Additionally, the Cu(II) ion in Hairvogine multivitamin has been effectively identified using the produced Cu(II) carbon paste electrode. A muffle furnace was used to heat three tablets in a silica crucible for seven hours at a temperature of roughly 650 °C. In a measuring flask, the resulting ash was then dissolved in 20 milliliters of HCl, followed by the addition of 100 milliliters of distilled water. NaOH or HNO_3_ was then used to adjust the pH to 4 at this point. Using the suggested sensor as an indication electrode and the conventional addition procedure, the resultant solution was utilized for Cu(II) ion analysis. Vegetable foliar fertilizer, namely Nutrifol (powder) was purchased from CHEMA Company. In order to determine the concentration of Cu(II) in it, a half gramme of the foliar was digested in 20 millilitres of distilled water by adding a few drops of strong HNO_3_. Prior to analysis, the pH was corrected to 4 and it was filtered and diluted to 100 mL with distilled water.

## Results and discussion

### Composition of carbon paste electrode

More than 70 distinct analytes, including inorganic and organic ions as well as certain nonanoic species including phenol derivatives and nonionic surfactants, can be quantified using ionophore-based ISEs^41^. The selective carbon paste electrode’s sensitivity, linear range, and amount of ionophore in the paste conformation are mostly influenced by the characteristics of the pasting liquid^42^. The electrode potential is caused by a reasonable chemical equilibrium that takes place at the electrode/solution boundary if there is an appropriate concentration of the used ionophore in the paste. On the other hand, excessive amounts of this material will alter the ionic sites ratio to the ionophore, which will result in poor performance^43^. After preparing and calibrating various electrodes with varying concentrations (5–20 mg) of 2-(((3-aminophenyl) imino) methyl) phenol Schiff base using the Cu(II) solution, the slope was discovered. According to Table 1’s slope, linear range, and regression, it was clear that 10 mg was the ideal ionophore concentration. Lipophilic additives (plasticisers) have been found to have a significant impact on the response of ion-selective electrodes due to their ability to increase selectivity, lower matrix resistance, improve response quality, and, in some cases, raise sensor sensitivity by improving the paste’s ability to extract the desired ions^17,44^. Five plasticizers, namely *o*-NPOE, TCP, DBP, DOP, and DHP were tested in sample electrodes as part of the search for an appropriate plasticizer in order to identify the one that responded the best. As a solvent arbitrator, TCP influences the ionophore distribution constant and liquifies things to enable paste homogeneity. TCP has every characteristic that makes a binder effective, including high conductivity and lipophilicity, low inclination to leach from the paste matrix, high capacity to dissolve the substrate and other paste additives, and non-volatility^45,46^.

**Table 1:** Carbon paste composition’s impact on electrode performance.

Electrode No.	Plasticizer (100 mg)	Schiff base Ionophore, mg	Graphite, mg	Slope ± SD*, mV decade^−1^	Linear range, mol L^−1^	*R* ^2^
1	TCP	0	250	18.14 ± 2.33	1 × 10^−7^- 1 × 10^−1^	0.8086
2	TCP	5	250	28.50 ± 1.12	1 × 10^−5^- 1 × 10^−1^	0.9975
3	**TCP**	**10**	**250**	**29.57 ± 0.80**	**1 × 10** ^**−7**^ **- 1 × 10** ^**−1**^	**0.9999**
4	TCP	15	250	29.50 ± 1.50	1 × 10^−4^ −1 × 10^−1^	0.9957
5	TCP	20	250	27.75 ± 2.78	1 × 10^−4^ −1 × 10^−1^	0.9918
6	DOP	10	250	31.60 ± 2.82	1 × 10^−5^- 1 × 10^−1^	0.9902
7	*o*-NPOE	10	250	21.80 ± 1.84	1 × 10^−5^- 1 × 10^−1^	0.9932
8	DHP	10	250	23.68 ± 7.39	1 × 10^−5^- 1 × 10^−1^	0.9518
9	DBP	10	250	25.40 ± 6.24	1 × 10^−5^- 1 × 10^−1^	0.9755

*SD of 5 replicates.

### Selectivity analysis

**Fig. 2:**
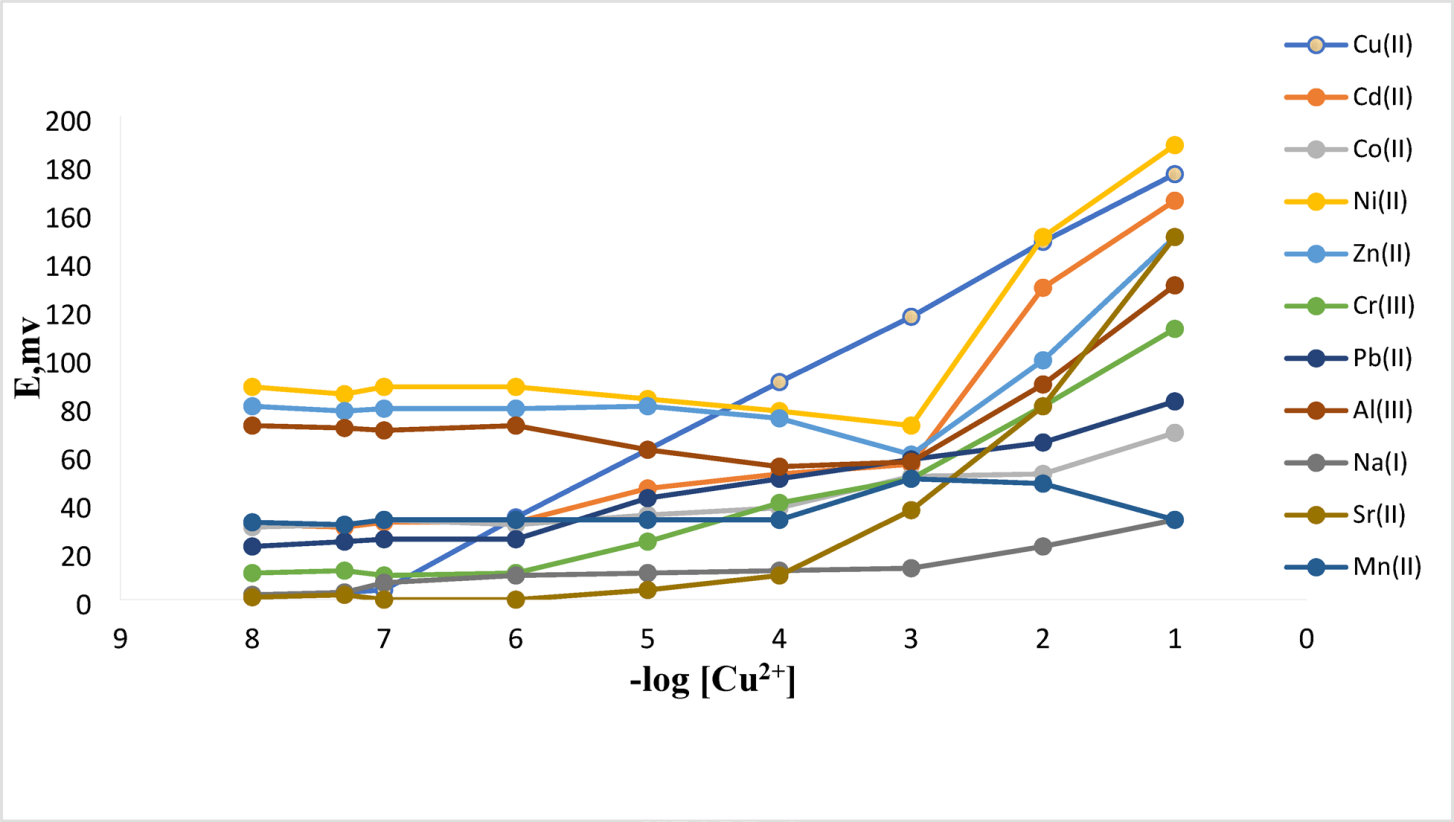
Change in sensor No. 3’s potentials for Cu(II) and other metal ions.

The primary regulator of an ISE membrane’s selectivity is the ionophore. The potentiometric ion sensors’ fundamental mechanism is established by the separation of two immiscible phases by ions the analyte and the ionophore attraction degree as well^16^. Concerning the production of graphite electrodes that possess the identical structure as electrode No. 3, included in Table 1, modification by Schiff base under study was applied. Figure 2 shows potential responses of the electrodes to different metal ions. Among all the cations that were investigated, it was discovered that the Cu(II) ion exhibited the Nernstian potential response throughout a broad concentration range. This is due to Cu(II) selective behaviour, enhanced ionophore interaction, and rapid exchange kinetics^16,47^. The selectivity coefficients of the recommended electrode towards different cationic species (M^n+^) were examined in this work using each of the fixed interference technique (FIM), the separate solution method (SSM) and matched potential method (MPM)^48,49^. SSM evaluates the selectivity coefficients by measuring potentials of other interfering ions and the fixed concentrations of Cu(II) ions separately using the following equation^47^:

1 $$\:log{{\:K}_{A}{,}_{B}\:}^{SSM}=\frac{{E}_{B}-{E}_{A}}{S}+\left(1-\frac{{Z}_{A}}{{Z}_{B}}\right)log\:{a}_{A}$$

where E_A_ and E_B_ were the measured potential of Cu(II) and interfering ions, respectively, z_A_ and z_B_ were charge numbers of the primary ion, A, and of the interfering ion, B, S was the slope and a_A_ was the activities of the primary ion, A. In Eq. (1) it is considered that a_A_=a_B_ and E_A_ and E_B_ were the response of the electrode to primary and interfering ions, respectively.

However, Cu(II) ions were present at varying concentrations in the fixed concentration of interfering ion in FIM and selectivity coefficient was calculated from the following equation^47^:

2 $$\:{{K}_{A}{,}_{B}\:}^{FIM}=\frac{{a}_{A}}{{\left({a}_{B}\right)}^{{z}_{A}/{z}_{B}}}$$

In Eq. (2), a_A_ was the primary ion activity at the detection limit and a_B_ was the interfering ion activity in the background.

In MPM, on the other hand, the ratio of primary and interfering ions under identical conditions that result in identical potential changes is used to calculate the selectivity coefficient of suggested sensors. A solution of fixed activity of primary ion is used as a reference solution (a_A_) and the first change in potential upon changing the primary ion activity (a_A_’) is measured, and then the interfering ion would be added to an identical reference solution until the same potential change is obtained. Then, the ratio between the activities of the primary ion A relative to the interfering ion B denotes the selectivity coefficient K^Pot^_A, B_ as shown in the following equation^48^:

3 $${{\text{K}}_{{\text{A}},{\text{B}}}}={\text{ }}({\text{a}}_{{\text{A}}}^{\prime }-{{\text{a}}_{\text{A}}}){\text{ }}/{\text{ }}{{\text{a}}_{\text{B}}}$$

The values of the selectivity coefficients are shown in Table 2. The suggested sensor does a respectable job of distinguishing Cu(II) ions from other metal ions, as evidenced by the selectivity coefficient values.

**Table 2:** Cu(II) sensor selectivity coefficients when additional ions are present.

Foreign ion	Log K ^SSM^_Cu (II), B_	Log K ^FIM^_Cu (II), B_	Log K ^MPM^_Cu (II), B_
Ni^2+^	−1.52	−2.60	−3.01
Cd^2+^	−2.06	−3.00	−2.59
Cr^3+^	−3.27	−4.82	−2.61
Co^2+^	−2.23	−3.00	−2.78
Mn^2+^	−2.27	−2.70	−2.90
Zn^2+^	−1.93	−2.80	−2.78
Sr^2+^	−2.60	−2.50	−2.69
Pb^2+^	−2.01	−4.11	−3.55
Sr^2+^	−2.71	−3.57	−3.74
Al^3+^	−3.03	−3.58	−2.78
Sn^2+^	−1.23	−2.49	−2.83
K^+^	−0.76	−1.29	−2.48
Na^+^	−0.52	−1.30	−2.85

### Effect of pH

**Fig. 3:**
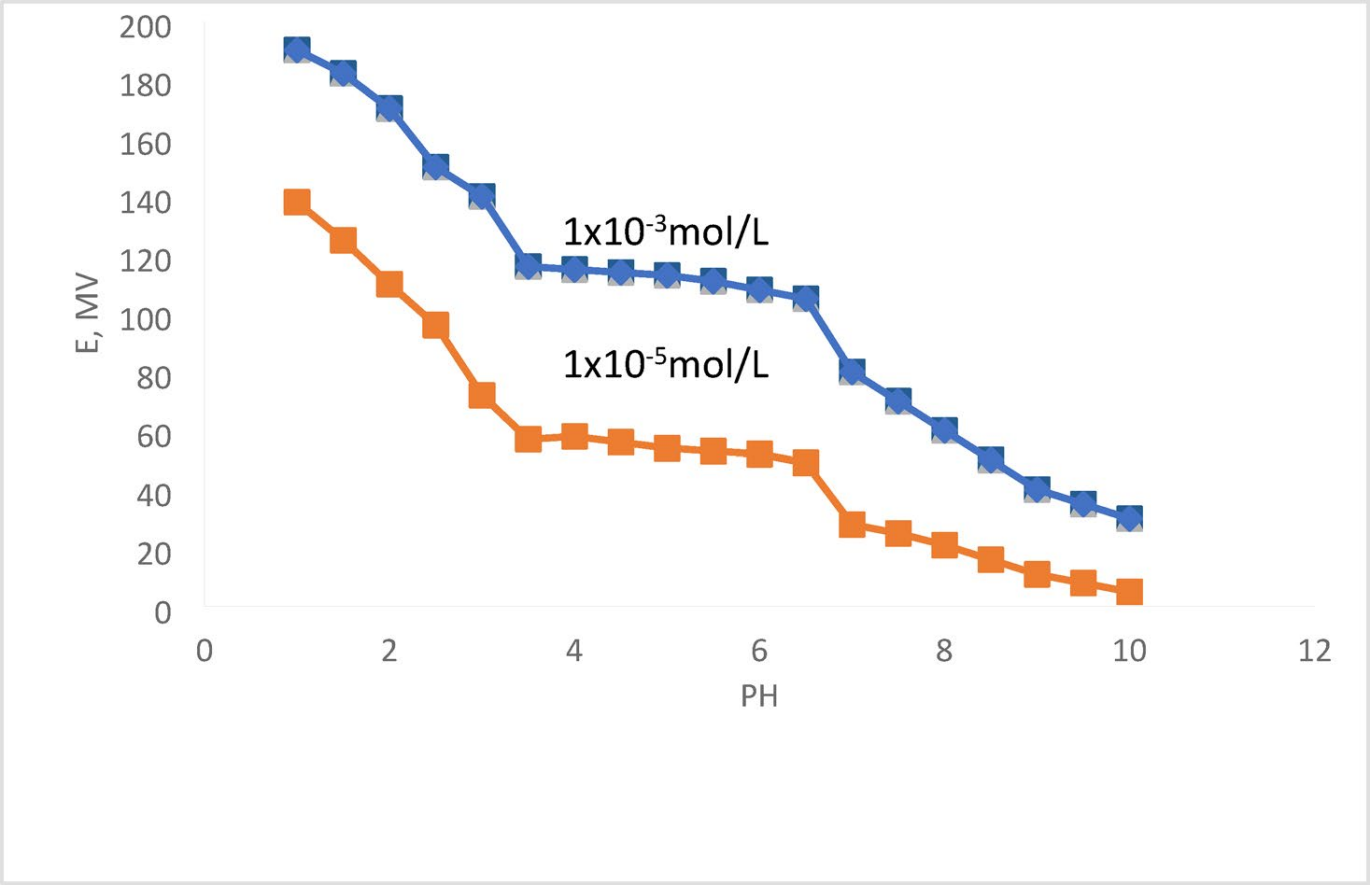
pH impact on the electrode performance.

The impact of pH on the functionality of the most sensitive electrode (No. 2) was examined over the pH range of 1.0–10.0 using 1.0 × 10^−3^ mol L^−1^ and 1.0 × 10^−5^ mol L^−1^ two disimilar Cu(II) concentrations. The findings are shown in Fig. 3. The varying of pH ranges from 3.5 to 6.5 caused the potential to stay constant. These findings were in line with those of other Cu(II) sensors^32–34^.

The pH was adjusted using 1.0 mol L^−1^ of HNO_3_ and NaOH. The substantial quantity of H_3_O^+^ ions that produced an interference may have been the reason for the potential shift at pH 3.5 because these ions at such high concentrations can induce interference via ligand protonation^16,33,34^. However, fluctuations in potential occur when pH rises above 6.5 due to the production of Cu(OH)_2_, which cannot react with the ionophore^34^.

### Response time and reversibility

**Fig. 4:**
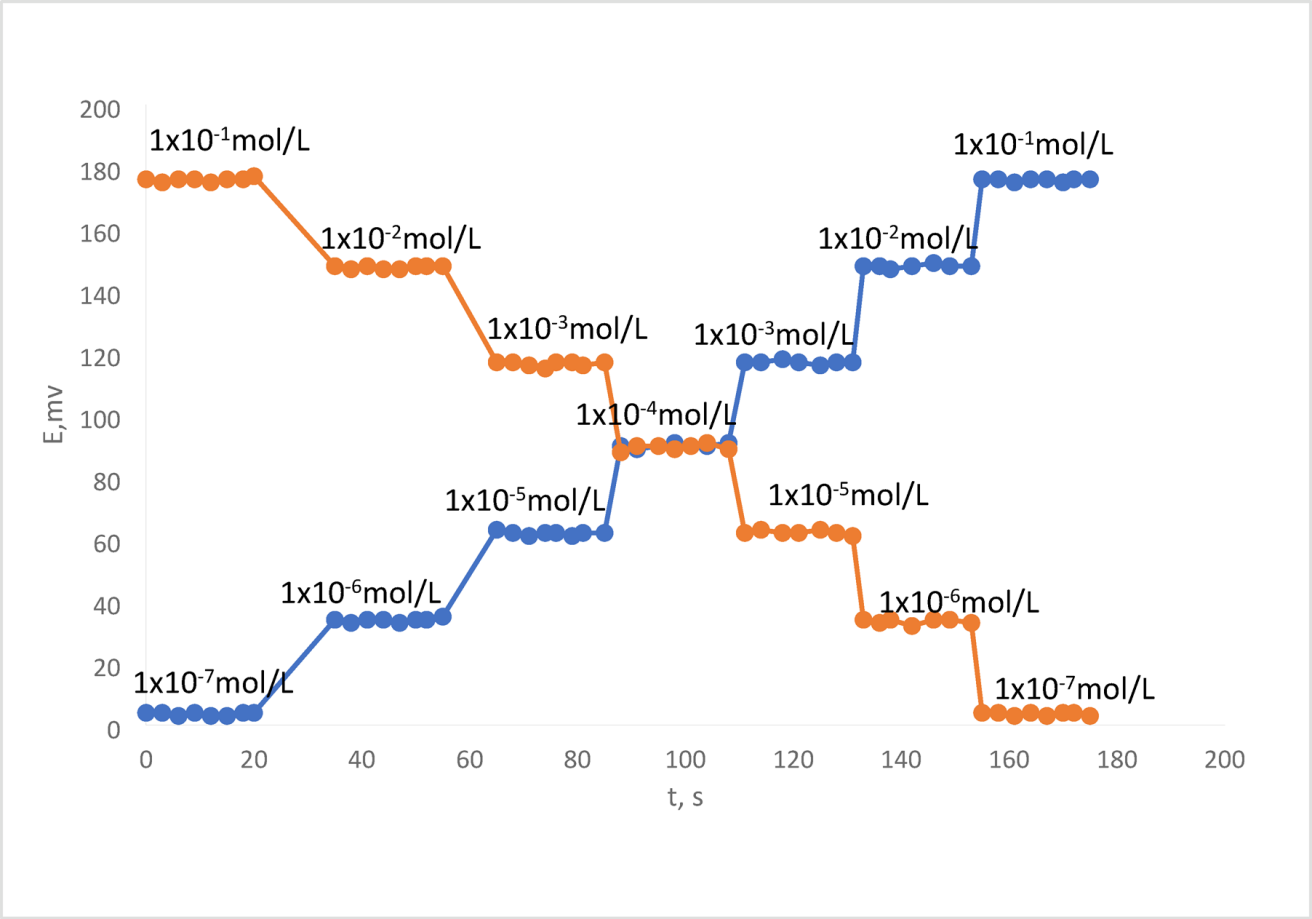
Response time and reversibility.

Response time, or the average time required for a sensor to reach a potential between + 1 and − 1 mV of the final equilibrium value, is the most crucial component of any sensor. The critical emf response of the electrode was assessed in accordance with IUPAC regulations^50^. It was discovered to be less than fifteen seconds, with a three-minute potential consistency^51^, Potential was tracked against time using Cu(II) sulphate solutions at different concentrations (Fig. 4). The findings in Fig. 3 demonstrated the reversibility of the potential response, suggesting that there was no discernible memory impact on the electrode response^34^.

### Effect of temperature

Calibration curves were built at various temperatures within the range of 20–70 °C to investigate the impact of temperature on the EMF response of the suggested sensor. Table 3 reveals that the suggested sensor’s calibration graph exhibited a Nernstian slope up to 60 degrees Celsius in the test solution, while the linear concentration range remained nearly constant. This implies that there will be no discernible departure from Nernstian behaviour when using the studied electrode up to 60 °C. However, temperatures beyond 60 °C produced a notable deviation from the anticipated results, which can be explained by some leaching in the paste matrix damaging the electrode surface and reducing responsiveness^52^. Plotting the standard cell potentials, E° cell, against (t – 25) at various temperatures was done using the corresponding calibration graphs. The intercept of these plots at -log [Cu(II), mol L^−1^] = 0 was used to derive dE/dt; the isothermal coefficient of the cell. Applying Antropov’s equation, where t was the temperature degree in °C^53–55^. The low value of the isothermal coefficient, 9.3 × 10^−5^ V °C^−1^, indicates that the tested sensor has strong thermal stability in the examined temperature scale.

**Table 3:** Temperature impact on the proposed Cu(II) sensor’s response.

T ºC	Slope (mV decade^−1^)	Linear range (mol L^−1^)	*R* ^2^
20	29.14	1 × 10^−7^– 1 × 10^−1^	0.9993
30	29.31	1 × 10^−7^– 1 × 10^−1^	0.9997
40	29.17	1 × 10^−7^– 1 × 10^−1^	0.9934
50	28.23	1 × 10^−7^– 1 × 10^−1^	0.9987
60	29.11	1 × 10^−7^– 1 × 10^−1^	0.9998
70	18.87	1 × 10^−6^– 1 × 10^−1^	0.9921

### Response mechanism and morphological studies

**Fig. 5:**
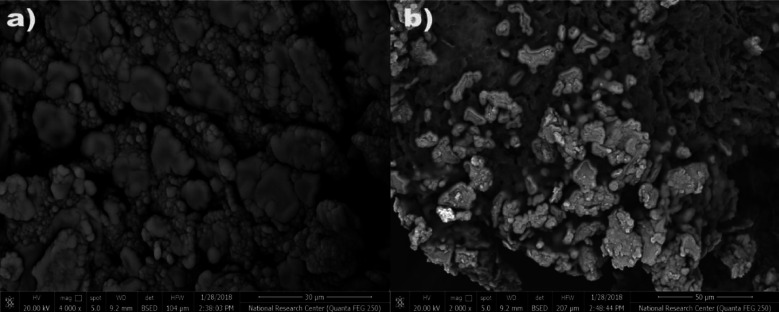
The suggested sensor’s surface (electrode no. 3) is shown in the SEM image (a) before and (b) after soaking in Cu(II) ion (1.0 × 10^−3^ mol L^−1^) for one hour at 25 °C.

**Fig. 6:**
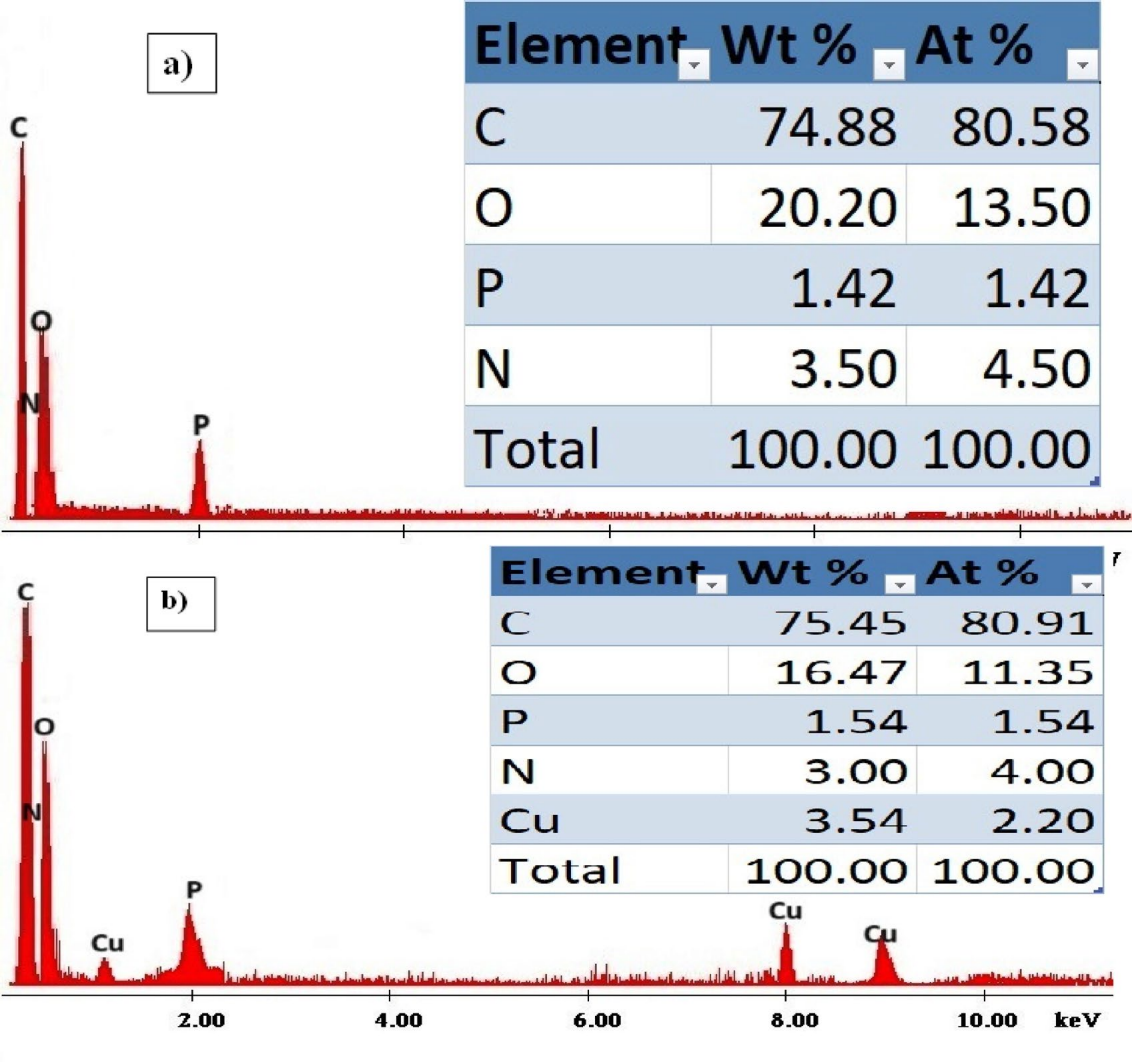
EDX analysis showing weight% of different elements present in the suggested sensor before soaking (a) and after soaking (b) in 1.0 × 10^−3^ mol L^−1^ Cu(II) solution for one hour at 25 °C.

**Fig. 7:**
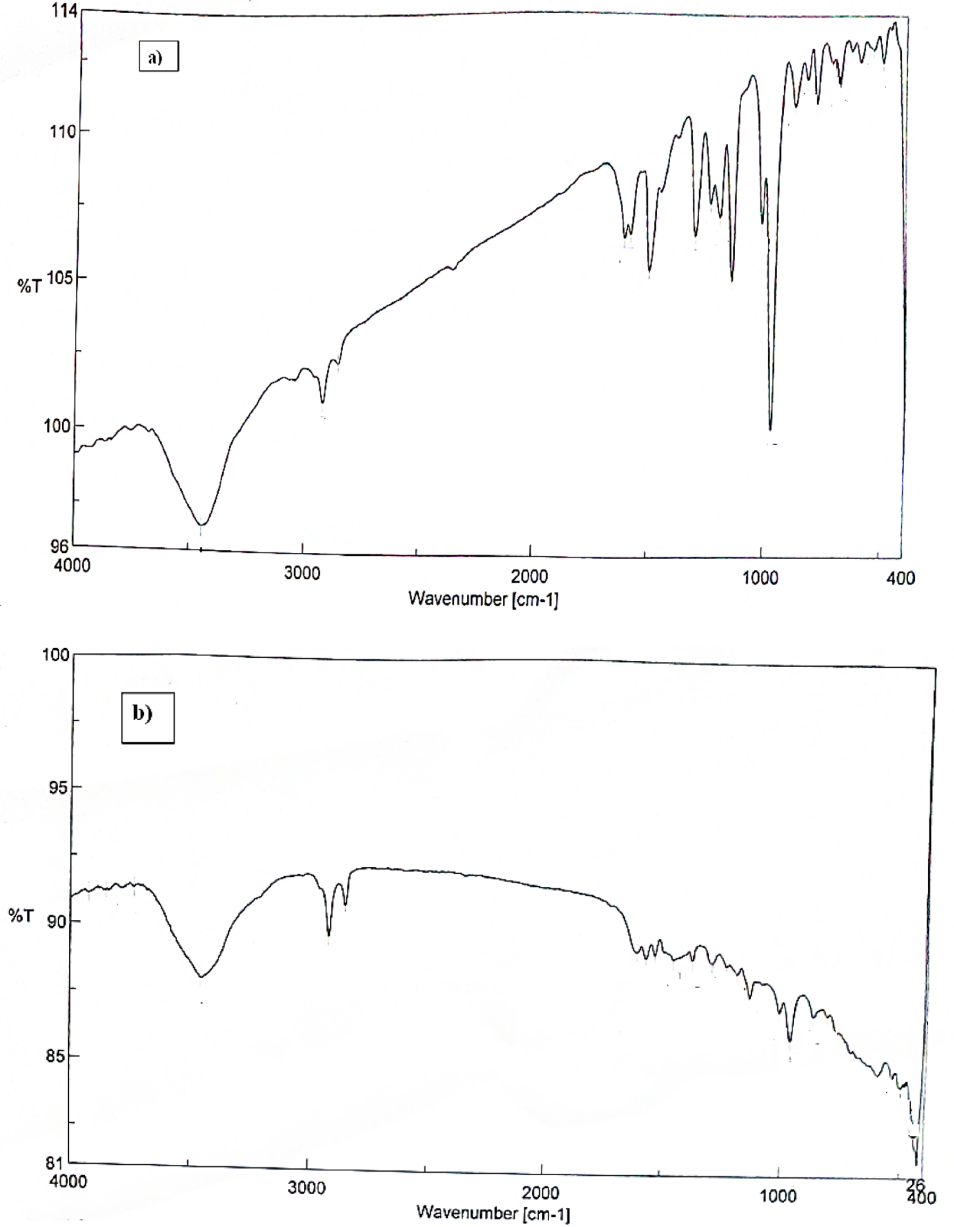
FT-IR spectra of the applied Schiff base in the MCPE (a) before and (b) after interaction with Cu(II) ion in 1.0 × 10^−3^ mol L^−1^ solution for one hour at 25 °C.

The degree to which the target ion may be extracted selectively determines the ISE performance. SEM and EDX, two methods that are deemed to be crucial for relating the sensor’s response to surface morphology, were applied to clarify the surface of the suggested electrode^16^. SEM was utilized to assess and validate this generated complex, in addition to EDX and IR studies. EDX and SEM were employed in an effort to link the potentiometric response to morphology^17^. The homogenous and permeable sensor surface made it easier for the Cu(II) ions to be extracted into the paste, where they subsequently manifested as illuminated spots inside the gaps between the graphite grains^34^ evident in the SEM image (Fig. 5a) and caused the surface morphology to change (Fig. 5b). EDX analysis corroborated this mechanism of Cu(II) ion insertion and complex formation; the elemental weight percentages on the paste surface before soaking in Cu(II) ion solution were C: 74.88, O: 20.20, P: 1.42, N: 3.50, while these percentages were changed after soaking in 1.0 × 10^−3^ mol L^−1^ solution into C: 75.45, O: 16.47, P: 1.54, N: 3.0 and Cu: 3.54 and it is obvious from the results that the Cu(II) ion entered into the carbon paste and interacted with the applied ionophore (Fig. 6a and b). The IR spectrum of the Schiff base ionophore (Fig. 7a and b) showed peak at 1613 cm^−1^ due to the azomethine group. This band was shifted to 1628 cm^−1^ indicating participation of the azomethine nitrogen in complexation to Cu(II) ion^38^. The ligand had bands at around 1274 and 3380 cm^−1^, which correspond to NH_2_ and phenolic OH, respectively. These bands were discovered in the Cu(II) complex IR spectra at 1239 and 3450 cm^−1^^38^. Following soaking in Cu(II) solution, the bands that emerged in the paste’s infrared spectrum at 539 and 461 cm^−1^ were attributed to the υ(M-O) and υ(M‐N) stretching vibrations, respectively^38^.

### Sensitivity, reproducibility and life time

**Fig. 8:**
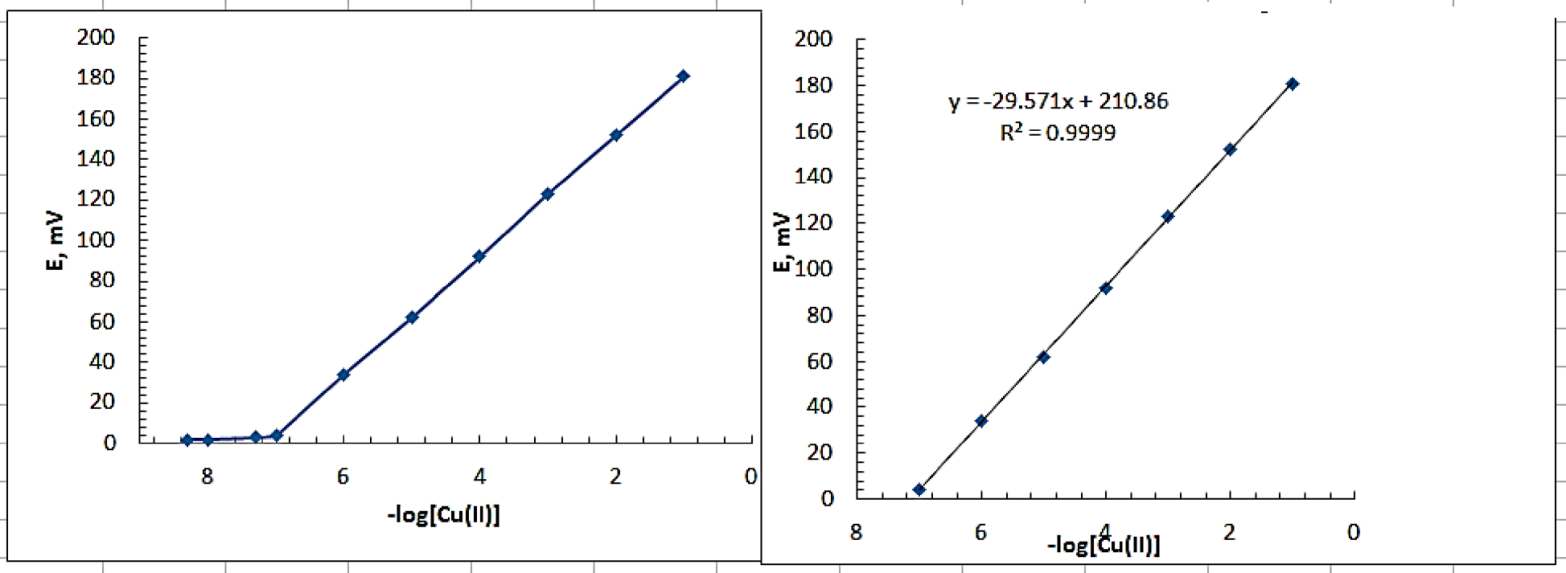
Calibration curve showing the response of the suggested MCPE towards Cu(II) ions.

In compliance with IUPAC guidelines, the limits of detection (LOD) and quantification (LOQ) were assessed in order to validate an analytical method, especially when identifying contaminants^56^. LOD, or lower limit of detection, is the lowest concentration of analyte in a sample that can be detected by it; it is not necessarily quantified as an exact amount. Conversely, the limit of quantification (LOQ) represents the lowest concentration of analyte in a sample that can be precisely and accurately quantified as a number. This is especially important when the intended analytical approach is to identify contaminants. Visual estimation of LOD can be done using the point where a calibration curve’s two linear components intersect, or the Nernstian and non-Nernstian portions of the calibration plot (Fig. 8); LOQ was calculated to be 3.3 the LOD value.

The LOD for the Cu(II) ion was determined to be 5.0 × 10^−8^ mol L^−1^, while the limit of quantification was 1.65 × 10^−7^ mol L^−1^. On the other hand, a set of five pastes with the optimal mix was made in order to evaluate the repeatability of the proposed sensor. The electrodes’ responses were evaluated for Cu(II) ion concentrations of (0.635 × 10^−3^, 6.350 × 10^−3^ and 63.50 × 10^−3^ mg mL^−1^) with RSD% value of 3.1, 4.4, and 3.69, respectively. The results showed that the recommended electrode was highly reproducible. Additionally, the intra- and inter-day studies on a range of sample concentrations were reported as given in Table 4. These results demonstrated that the Cu(II) sensor’s recommended level of accuracy and repeatability to be sufficient. In terms of the sensor’s lifetime, consistent and reliable signals were get for 2 months (wherein the sensor was actively utilised for one hour each day). The deviation from Nernstian behavior and reduction in sensitivity could be attributed to the mechanical stability of the paste deteriorating over time. But if the electrode is stored in distilled water in case of non-use, its lifespan can be increased by many months.

**Table 4:** Reproducibility of the suggested sensor.

Taken (mg ml^−1^)	Intra-day				Inter-day			
	Found (mg ml^−1^)	Recovery %	SD*	RSD %	Found (mg ml^−1^)	Recovery %	SD*	RSD %
0.635 × 10^−3^	0.606 × 10^−3^	95.43	1.14 × 10^−6^	1.23	0.622 × 10^−3^	97.95	4.04 × 10^−7^	2.12
6.35 × 10^−3^	5.90 × 10^−3^	92.91	1.48 × 10^−5^	2.08	6.500 × 10^−3^	102.36	5.77 × 10^−6^	1.15
63.5 × 10^−3^	64.10 × 10^−3^	100.94	7.4 × 10^−5^	1.75	64.70 × 10^−3^	101.89	6.43 × 10^−5^	0.94

*SD of 6 replicates.

### Analytical application

The modified CPE under research was used to the analysis of the concentration of Cu(II) in various vegetable foliar, pharmaceutical and water samples in order to determine its applicability. Using an actual water sample with different amounts of other cations, the suggested electrode was used to measure the various spiking Cu(II) ions concentrations by direct calibration method. The recovery % has been shown to be satisfactory even in the presence of other cations, which may be related to the strong selectivity and low detection limit of the synthesized Cu(II) sensor. Table 5 provides a summary of the picked-up results. Furthermore, the utility of the sensor No. 3 was investigated to estimate Cu(II) ion in hairvogine multivitamin and vegetable foliar by standard addition mode. The results were close to that obtained by AAS and hence that sensor can be in use for Cu(II) quantification in real testers. The F-test was made to relate the statistical significance between the two techniques (AAS and CPE) and t-test was made to compare the means of the two techniques^9^.

**Table 5:** Measurement of Cu(II) in different real samples.

Samples	Taken, mg mL^−1^	Found,			RSD%*		Recovery %		t-test	F-test
		mg ml^−1^								
		Proposed carbon paste electrode		AAS	Proposed carbon paste electrode	AAS	Proposed carbon paste electrode	AAS		
Water sample (1)	0.635 × 10^−3^	0.633 × 10^−3^		0.631 × 10^−3^	2.05	1.89	99.69	99.37	0.254	1.193
	6.350 × 10^−3^	6.501 × 10^−3^		6.330 × 10^−3^	1.74	1.82	102.36	99.68	2.37	1.039
	63.50 × 10^−3^	64.20 × 10^−3^		63.47 × 10^−3^	2.61	2.36	101.1	99.95	0.726	1.252
Water sample (2)	0.635 × 10^−3^	0.631 × 10^−3^		0.632 × 10^−3^	1.78	1.49	99.37	99.53	0.153	1.422
	6.35 × 10^−3^	6.29 × 10^−3^		6.31 × 10^−3^	1.59	1.97	99.05	99.37	0.28	1.546
	63.50 × 10^−3^	63.45 × 10^−3^		63.44 × 10^−3^	0.98	0.95	99.92	99.91	0.026	1.064
Vegetable foliar	8.26	7.99		8.01	0.931	0.787	96.73	96.97	0.46	1.38
Hairvogine Multivitamin	0.03	0.0298		---------	0.78	-------	99.33	---------	----------	---------
Components of hairvogine multivitamin										
			Per 2 tablets							
Vitamin D (as D3 800 IU)			20 µg							
Vitamin E			40 mg a-TE							
Vitamin C			80 mg							
Thiamin (Vitamin B1)			8 mg							
Riboflavin (Vitamin B2)			4 mg							
Niacin (Vitamin B3)			18 mg NE							
Vitamin B6			10 mg							
Folic Acid			400 µg							
Vitamin B12			20 µg							
Biotin			150 µg							
Pantothenic Acid			40 mg							
Magnesium			75 mg							
Iron			14 mg							
Zinc			15 mg							
Copper			1000 µg							
Manganese			2 mg							
Selenium			165 µg							
Chromium			40 µg							
Iodine			200 µg							
Marine Collagen			200 mg							
L-Cystine			100 mg							
Inositol			200 mg							
L-Methionine			50 mg							
Coenzyme-Q10			5 mg							
Natural mixed caroienoids			2 mg							

*At confidence level 95% (*n* = 5), F-test (tabulated) = 5.192 and t-test (tabulated) = 2.571.

### Comparative study

The key attributes of performance (ionophore, slope, linear range, detection limit, pH, shelf and response time) were listed in Table 6 for the results of the suggested Cu(II) ISE versus the data of some cupper-selective electrodes from the literature^9,12,32–37,57,58^. The foundation of this work is the graphitic potentiometric electrode, which is simple, inexpensive, and renewable. In addition, it’s superior over some listed PVC membrane electrodes^9,12,33,37^ which suffers from increased system impedance and the electrode response time due to the presence of internal reference solution. Moreover, the lifetime of them will be shorter as a result of the electroactive material leaching throughout both solutions in contact with the membrane and finally due to the internal compartment, they couldn’t with stand high pressure. It is clear that this electrode is better than previously reported electrodes in most cases because it can be used in a wider concentration range, has rapid response time, and has improved sensitivity and selectivity for Cu(II) ions from a wide variety of other heavy metal ions (which is more appropriate for water, industrial and pharmaceutical samples). It must be noted that, there are voltammetric sensors^29^ that showed an enhancement in selectivity and sensitivity in determination of Cu(II) ions, but they are to some extent expensive and complicated. The goal of our work was to provide a simple, sensitive, reproducible and selective potentiometric Cu(II) sensor that can be applied widely in environmental, industrial, or pharmaceutical samples.

**Table 6:** Comparison between the proposed sensor and previously published sensors.

Reference	Ionophore				Slope, mV decade^−1^		Linear rang, mol L^−1^	Detection limit, mol L^−1^	pH	Life time	Response time (s)
^9^	Neutral carrier porphyrin				29.3		4.4 × 10^−6^–1.0 × 10^−1^	4.4 × 10^−6^	2.8–7.9	4 months	8
^12^	Dimethyl4,4′-(o-phenylene)bis(3-thioallophanate)				30.3		9.8 × 10^−6^–1 × 10^−1^	-	3.1–7.6	2 months	20
^32^	N, N0-bis(salicylaldehyde)-p-phenylene diamine (SPD)				30.12		1.0 × 10^−6^ −1.0 × 10^−2^	1.0 × 10^−6^	3.0–7.0	145 days	9
^33^	3-(4-nitrophenylazo)-pentane-2,4-dione				28.23		1.0 × 10^−6^–1.0 × 10^−1^	7.0 × 10^−7^	3.0–6.0	2 months	15
^34^	CPE I: dihydroxy anthraquinone (DHAQ) CPE II: DHAQ and Graphene CPE III: DHAQ and MWCNTs				I: 29.78 II: 30.25 III: 30.55		I and II: 1.0 × 10^−6^ – 1.0 × 10^−1^ III: 1.0 × 10^−5^ – 1.0 × 10^−1^	I: 8.0 × 10^−7^ II: 5.0 × 10^−7^ III: 3.3 × 10^−6^	2.4–6.5	I: 40 days II: 37 days III: 30 days	I: 10 II: 6 III: 3
^35^	2–furaldehyde thiosemicarbazone				28.5		1.0 × 10^−5^−1.0 × 10^−1^	6.89 × 10^−6^	5.0–9.0	-	5
^36^	5,5′-(1,4-phenylene)bis(1,3,4-thiadiazol-2-amine) (4)				32.3		1.0 × 10^−6^−1.0 × 10^−1^	9.02 × 10^−7^	4.0 − 9.0	-	-
^37^	1-Phenyl-2-(2-hydroxyphenylhydrazo)butane-1,3-dione (H2L)				28.80		2.0 × 10 − ^6^ −5.0 × 10^−3^	6.30 × 10^−7^	3.0–8.0	9 weeks	10
^57^	4-(2-(2,4-Dinitrophenylhydrazono) Methyl)Benzene-1,3-diol (L)				29.1		5.3 × 10^−8^ −1.0 × 10^−1^	2.1 × 10^−8^	3.1–8.2	2 months	9
^58^	Cd-MOF*				30.15		1.0 × 10^−7^- 1.0 × 10^−1^	7.5 × 10^−8^	2.5–6.5	2 months	10
This study	2-(((3-aminophenyl) imino) methyl) phenol				29.57		1 × 10^−7^- 1 × 10^−1^	5.0 × 10^−8^	3.5–6.5	2 months	< 15

MOF: Metal organic framework.

## Conclusion

The development of a novel Cu(II) ion selective carbon paste electrode involved the straightforward addition of 2-(((3‐aminophenyl) imino) methyl) phenol as an ionophore. Comparing the suggested sensor to the other previously published electrodes, it was discovered that the Cu(II) ISE performed better. Good Nernstian slope, low detection limit, accuracy and repeatability of this promising sensor make it to be considered as a good addition to the existing literature. This straightforward, affordable, and reusable Cu(II) sensor might be used in actual water, pharmaceutical and agricultural samples in a sensitive and focused manner for Cu(II) analysis.

## Data Availability

The datasets used and/or analysed during the current study are available from the corresponding author on reasonable request.

## Abbreviations

CPE: Carbon paste electrode
DBP: Dibutyl phthalate
DHP: Diheptyl phathalte
DOP: Dioctyl phthalate
EDX: Energy dispersive X-ray
FIM: Fixed interference method
ISE: Ion selective electrode
LOD: Limit of detection
LOQ: Limit of quantification
MCPE: Modified carbon paste electrode
MPM: Matched potential method
o-NPOE: o-Nitrophenyl octyl ether
SEM: Scanning electron microscope
SSM: Separate solution method
TCP: Tricresyl phosphate
